# Age and sex specific prevalences of cerebral β-amyloidosis, tauopathy and neurodegeneration among clinically normal individuals aged 50-95 years: a cross-sectional study

**DOI:** 10.1016/S1474-4422(17)30077-7

**Published:** 2017-04-26

**Authors:** Clifford R. Jack, Heather J. Wiste, Stephen D. Weigand, Terry M. Therneau, David S. Knopman, Val Lowe, Prashanthi Vemuri, Michelle M. Mielke, Rosebud O. Roberts, Mary M. Machulda, Matthew L. Senjem, Jeffrey L. Gunter, Walter A. Rocca, Ronald C. Petersen

**Affiliations:** 1 Department of Radiology, Mayo Clinic, 200 First Street SW, Rochester, MN, 55905; 2 Health Sciences Research, Mayo Clinic, 200 First Street SW, Rochester, MN, 55905; 3 Neurology, Mayo Clinic, 200 First Street SW, Rochester, MN, 55905; 4 Nuclear Medicine, Mayo Clinic, 200 First Street SW, Rochester, MN, 55905; 5 Psychiatry and Psychology, Mayo Clinic, 200 First Street SW, Rochester, MN, 55905; 6 Information Technology, Mayo Clinic, 200 First Street SW, Rochester, MN, 55905

## Abstract

**Background:**

A new descriptive classification scheme for biomarkers used in Alzheimer's and cognitive aging research, labeled ATN, was recently proposed. One implementation of this ATN construct dichotomizes biomarkers of amyloid, tau, and neurodegeneration/neuronal injury as normal or abnormal resulting in 2 × 2× 2=8 possible biomarker profiles. We determined the clinical characteristics and prevalence of each ATN group among clinically normal individuals aged 50 and older from a population based cohort.

**Methods:**

All individuals in this study were participants in the Mayo Clinic Study of Aging, a population-based study of cognitive aging. Potential participants were randomly selected from the Olmsted County, Minnesota population by age- and sex-stratification and invited to participate in cognitive evaluations and undergo multimodality imaging. To be eligible for inclusion in this study, participants must have been judged clinically to have no cognitive impairment and have undergone multi-modality imaging. Imaging studies were obtained from October 11, 2006 to October 5, 2016. All participants were classified as having normal (A−) or abnormal (A+) amyloid using amyloid PET, normal (T−) or abnormal (T+) tau using tau PET, and normal (−) or abnormal (N+) neurodegeneration/neuronal injury using cortical thickness. The cut points used were SUVR 1·42 (centiloid 19) for amyloid PET, 1·23 SUVR for tau PET, and 2·67 mm for MRI cortical thickness. Age- and sex- specific prevalences of the eight ATN biomarker groups were determined using 435 individuals with amyloid PET, tau PET, and MR imaging and 1113 additional clinically normal individuals who underwent amyloid PET and MR imaging, but not tau PET imaging.

**Findings:**

There were 165 A−T−N-, 35 A−T+N-, 63 A−T−N+, 19 A−T+N+, 44 A+T−N−, 25 A+T+N−, 35 A+T−N+, and 49 A+T+N+ individuals. Age differed by ATN group (p<0 001) ranging from a median age of 57 in the A−T−N-−and A−T+N− groups to 80 in the A+T−N+ and A+T+N+ groups. The frequency of APOE ε4 carriers differed by ATN group (p=0·04) with ε4 carriers roughly twice as frequent in A+ versus A−. White matter hyperintensity volume (p<0·0001), and cognitive performance (p<0·0001) also differed by ATN group. Tau PET and neurodegeneration biomarkers were discordant in the majority of individuals who would be labeled stage 2/3 preclinical AD (86% at age 65 and 51% at age 80) or suspected non-Alzheimer's pathophysiology (SNAP) (92% at age 65 and 78% at age 80). From age 50, A−T−N− prevalence declines while A+T+N+ and A−T+N+ increase continuously with age. In both men and women, A−T−N− is the most prevalent group until their late 70s. After about age 80, A+T+N+ is the most prevalent group until their late 70s. After about age 80, A+T+N+ is the most prevalent group. The remaining ATN groups reach individual peaks in the 60–90 age range and then decline in prevalence. By age 85 over 90% of men and women have one or more biomarker abnormalities.

**Interpretation:**

Biomarkers of fibrillar tau deposition can be included with those of Aβ and neurodegeneration/neuronal injury to more fully characterize the heterogeneous pathological profiles in the population. The prevalence of each ATN group changes substantially with age with progression toward more biomarker abnormalities even among individuals who remain clinically normal. Both abnormal amyloid and normal amyloid pathological profiles can be identified in the clinically normal population.

## Introduction

Use of biomarkers as an aid in the diagnosis of Alzheimer's disease (AD) gained acceptance with the publication of the National Institute on Aging - Alzheimer's Association (NIA-AA) recommendations ^1-4^ and the International Working Group (IWG) criteria ^5,6^ for AD.^1^ In the NIA-AA recommendations biomarkers were divided into two classes: biomarkers of amyloid (A) and biomarkers of tau-related neurodegeneration/neuronal injury (N).^1, 21, 25, 6^When the NIA-AA preclinical AD staging recommendations were operationalized and applied to a cohort of 450 clinically normal (CN) individuals over age 70, roughly one third fell into stages 1–3 of preclinical AD, 40% were amyloid normal and neurodegeneration normal (A− N−), and one quarter were amyloid normal and neurodegeneration abnormal (A− N+)^7.^We labeled the A− N+ group suspected non- Alzheimer's pathophysiology (SNAP) on the assumption that this was a pathologically heterogeneous group with a variety of non-Alzheimer's pathologies. To reflect NIA-AA staging while accounting for SNAP and A­N− groups, many research groups have adopted a 2-class biomarker construct in which individuals are assigned to one of four biomarker categories: A− N−, A+N−, A− N+ (SNAP) or A+N+. ^8-13^ This approach has been useful because it provided a common framework for different research groups to communicate findings in their own cohorts.^14-18^

In retrospect, a weakness of the NIA-AA staging plus SNAP construct was the grouping of CSF phosphorylated tau, MRI and FDG PET into one neurodegeneration/neuronal injury category. ^19 1,^ n persons with AD it is reasonable to assume that neurodegeneration in AD-sensitive areas is most often related to tauopathy; however, neurodegeneration, even when defined based on its pattern in AD, also occurs in non-AD conditions. A solution to this problem is to separate biomarkers that are specific for fibrillar tau deposits and its associated pathophysiology from those that are nonspecific measures of neurodegeneration/neuronal injury. This refinement enables identification of tauopathy and neurodegeneration/neuronal injury that are and are not associated with each other, leading to a more precise understanding of the biological underpinnings of brain aging. To this end an international group recently proposed a new descriptive classification scheme^20^ for biomarkers used in AD and cognitive aging research. The construct is labeled ATN^20^ and is based on grouping biomarkers into three categories: fibrillary β-amyloid deposition or associated pathophysiology (A);^19^ paired helical filament tau or associated pathophysiology (T);^14-19^ and, neurodegeneration or neuronal injury (N). One possible implementation of ATN is to dichotomize each biomarker category as either normal (−) or abnormal (+) which results in 2×2×2=8 different biomarker group combinations.

The goal of the current study was to apply the ATN categorization to clinically normal (CN) individuals aged 50 and older in the population-based Mayo Clinic Study of Aging to estimate the age and sex-specific prevalences of each ATN group and to describe the clinical and demographic characteristics of the eight ATN biomarker groups. We used amyloid PET to define A, tau PET to define T, and cortical thickness to define N.

## Methods

### Study design and participants

All individuals in this study were participants enrolled in the Mayo Clinic Study of Aging (MCSA), a population-based study of cognitive aging among Olmsted County, Minnesota residents ^21^ The Rochester Epidemiology Project ^22^ medical records linkage system was used to enumerate all Olmsted County residents aged 50 to 89. Potential participants were randomly selected from this enumeration according to age and sex strata with equal numbers of men and women in each age category. All individuals without a medical contraindication are invited to participate in imaging studies. Since 2004, the MCSA has enrolled non-demented individuals aged 70 to 89 years, and in 2012 started to enroll subjects 50 plus years of age. ^7, 8, 21^ Prior to May 28, 2015 imaging included amyloid PET, FDG PET, and MRI. Beginning May 28, 2015 individuals who participated in imaging underwent each of amyloid PET, tau PET, and MR imaging. ^21^

Individuals from the MCSA were included in the current cross-sectional study if they were judged clinically to have no cognitive impairment and have undergone amyloid PET, tau PET (in a subset), and MR imaging between October 11, 2006 and October 5, 2016. We analyzed data from the first visit with amyloid PET, tau PET, and MRI or the more recent amyloid PET and MRI visit if no tau PET was available to estimate the age and sex-specific prevalences of each ATN group and to describe the clinical and demographic characteristics of the eight ATN biomarker groups

The MCSA and related studies were approved by the Mayo Clinic and Olmsted Medical Center Institutional Review Boards and written informed consent was obtained from all participants

### Procedures

Amyloid PET imaging was performed with Pittsburgh Compound B^19^, synthesized on site with precursor purchased from ABX Biochemical Compounds, Germany. Tau PET was performed with AV1451, synthesized on site with precursor supplied by Avid Radiopharmaceuticals ^17^. Late uptake amyloid PET images were acquired from 40-60 minutes and tau PET from 80-100 minutes after injection. Methods of amyloid PET data analysis have been described previously. ^7,23^ Amyloid PET values are expressed both in SUVR units and in centiloid units.^24^ A tau PET composite reporter region of interest (ROI) was formed from a voxel-number weighted average of the median uptake in the entorhinal, amygdala, parahippocampal, fusiform, inferior temporal, and middle temporal ROIs normalized to the cerebellar crus grey median. ^23^ PET data was not partial volume corrected.

MRI was performed on one of three 3 Tesla systems from the same vendor (General Electric, Waukesha WI, USA). The primary MRI measure was a FreeSurfer (v5·3) derived temporal lobe cortical thickness composite reporter ROI of the entorhinal, inferior temporal, middle temporal, and fusiform ROIs. ^23^ These were consistently among the top performing ROIs across our previous ROI selection studies discriminating between A– clinically normal and A+ impaired individuals. ^25, 26^ As an alternative measure of neurodegeneration we used the sum of right and left hippocampal volumes from FreeSurfer adjusted for total intracranial volume (HVa) as described in ^27^. The MRI acquisition also included a FLAIR sequence from which white matter hyperintensity volume was measured using an algorithm developed in-house.^28^

We have recently conducted a thorough examination of several different methods for selecting cut-points to define abnormality on amyloid PET, tau PET and MRI thickness. ^23^ The optimal amyloid PET cut-point of SUVR 1·42 (centiloid 19) was based on the threshold value beyond which the rate of change in amyloid PET reliably increases. We determined cut-points for tau PET and MRI thickness by maximizing the accuracy (i.e., maximizing sensitivity plus specificity) in discriminating between amyloid positive individuals with mild cognitive impairment or dementia versus MCSA CN individuals aged 30-49. Based on this method, the cut­point for tau PET was 1·23 SUVR and for MRI cortical thickness was 2·67 mm. Each participant in the present study was classified into one of the eight ATN states using these cut-points. As a secondary analysis, abnormal N was defined as HVa less than -1·15 cm^3^. This HVa cut-point was derived in the same manner and using the same samples described in. ^23^

### Statistical methods

**Figure 1:**
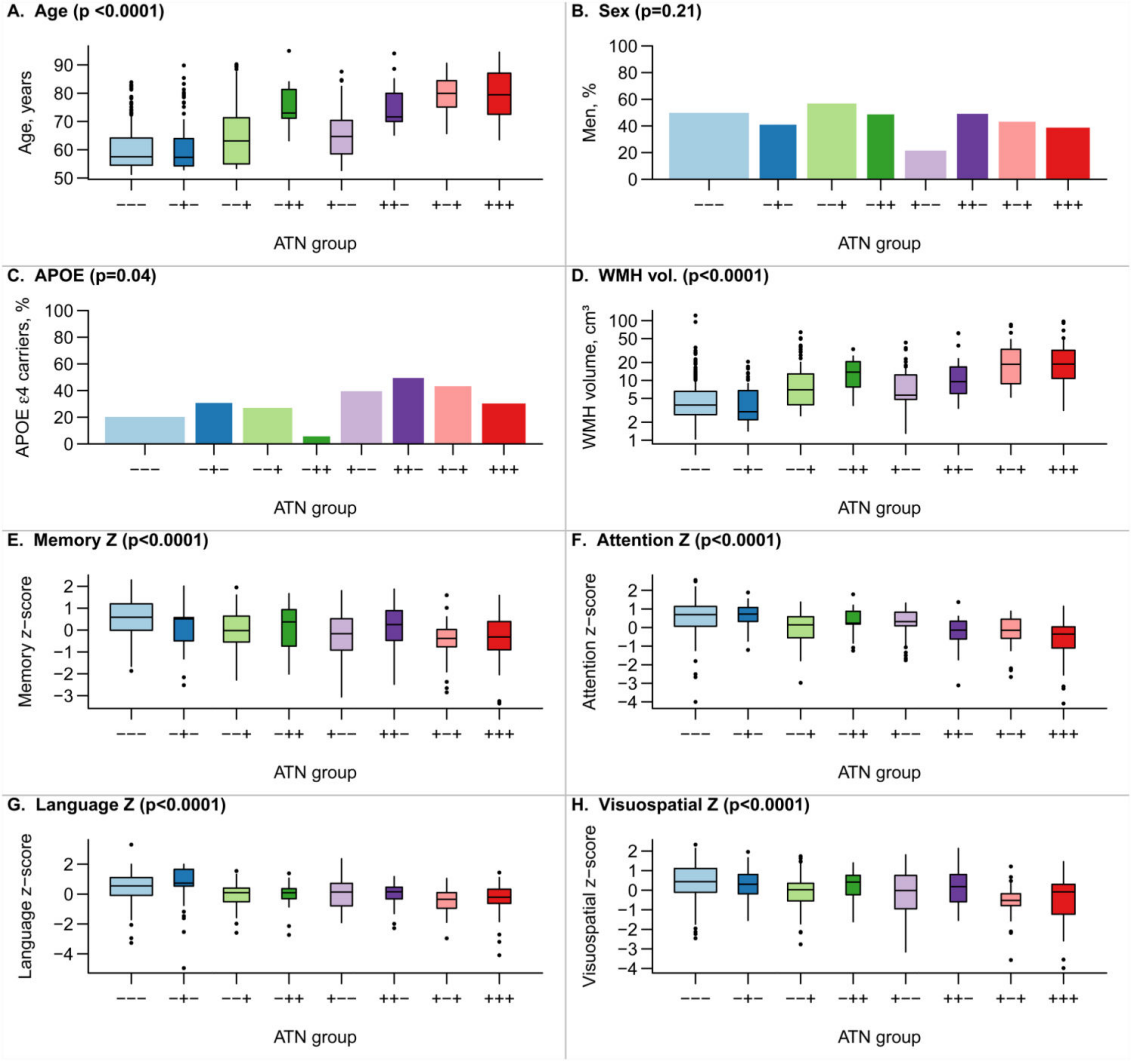
Plots of ATN group characteristics Box plots of continuous variables and bar charts summarizing percentages of categorical variables from table 1 by ATN biomarker group. The box plots and estimated percentages reflect weighting the sample to match the age and sex distribution of Olmsted County, Minnesota residents who are clinically normal. Box and bar widths reflect relative sample sizes. As in Table 1, the 8 groups are sorted left-right hierarchically first on the basis of A- vs A+, then T- vs T+, then N- vs N+.

The MCSA sampled similar numbers of subjects within 5-year age and sex strata from age 50-90. As a result, individuals in the older age strata were overrepresented relative to the population. Therefore to summarize the overall clinical and demographic characteristics of the eight ATN groups (Fig 1, S1), it was necessary to weight our sample to reflect the actual age and sex distribution of the Olmsted County, Minnesota clinically normal population. Census Bureau estimates for 2010 along with MCI and dementia prevalence estimates from the MCSA were used to create the weights and the *survey* package in R was used to correct standard errors to account for strata weights (see statistical supplement).

The estimated prevalence of each of the eight ATN groups was determined by partitioning the full 8-group model into two components: (1) a multinomial model with the 4-level AN group as the response and age and sex as covariates (n=1548) and (2) a logistic model with T+ as the response and AN, age, and sex as covariates (n=435). In this framework, the individuals without tau imaging can stabilize the overall estimates of the ATN prevalences by contributing information to part (1) of the model. Inference from the model was based on posterior simulations using the maximum likelihood estimate and the variance covariance matrix. These simulations allowed us to obtain point estimates and 95% confidence intervals for functions of the model parameters such as prevalence estimates, differences in prevalence estimates, and the age at which a prevalence curve peaks (see statistical supplement).

### Role of the funding source

The funders of the study had no role in study design, data collection, data analysis, data interpretation, or writing of the report. All authors had full access to all the data in the study. The corresponding author had final responsibility for the decision to submit for publication.

## Results

The data in Table 1 represent unweighted summaries in our ATN sample (n=435). Summaries by ATN group weighted to the clinically normal Olmsted County population by age and sex are found in Fig 1. Age differed among ATN groups (p<0·0001) with individuals with worse biomarker profiles tending to be older (Table 1, Fig 1a). The group with the greatest estimated proportion of men is A−T−N+ (57%, 95% CI: 37%-77%) and the group with the greatest proportion of women is A+T−N− (78%, 95% CI: 64%-93%) however overall the sex distribution was not different among the ATN groups (p=0·21). APOE ε4 varies by ATN group (p=0·04) and is roughly twice as frequent among A+ individuals compared to A− individuals. WMH volume differed between ATN groups (Fig 1, p<0·0001) even after adjustment for age (p=0·01) (Fig S1). WMH was higher in N+ compared to N− groups (p=0·05) (Fig 1, Fig S1, table 1), although the magnitude of the differences was small. Cognitive performance also differed to some degree by group in all domains (Fig 1, p<0·0001) even after adjustment for age (Fig S1, p<0·03).

Table S1 shows demographic features of the 1548 clinically normal MCSA individuals with amyloid PET and MRI but not tau PET that were used to constrain or stabilize ATN prevalence estimates among the subset of 435 who had amyloid PET, MRI and tau PET.

**Figure 2:**
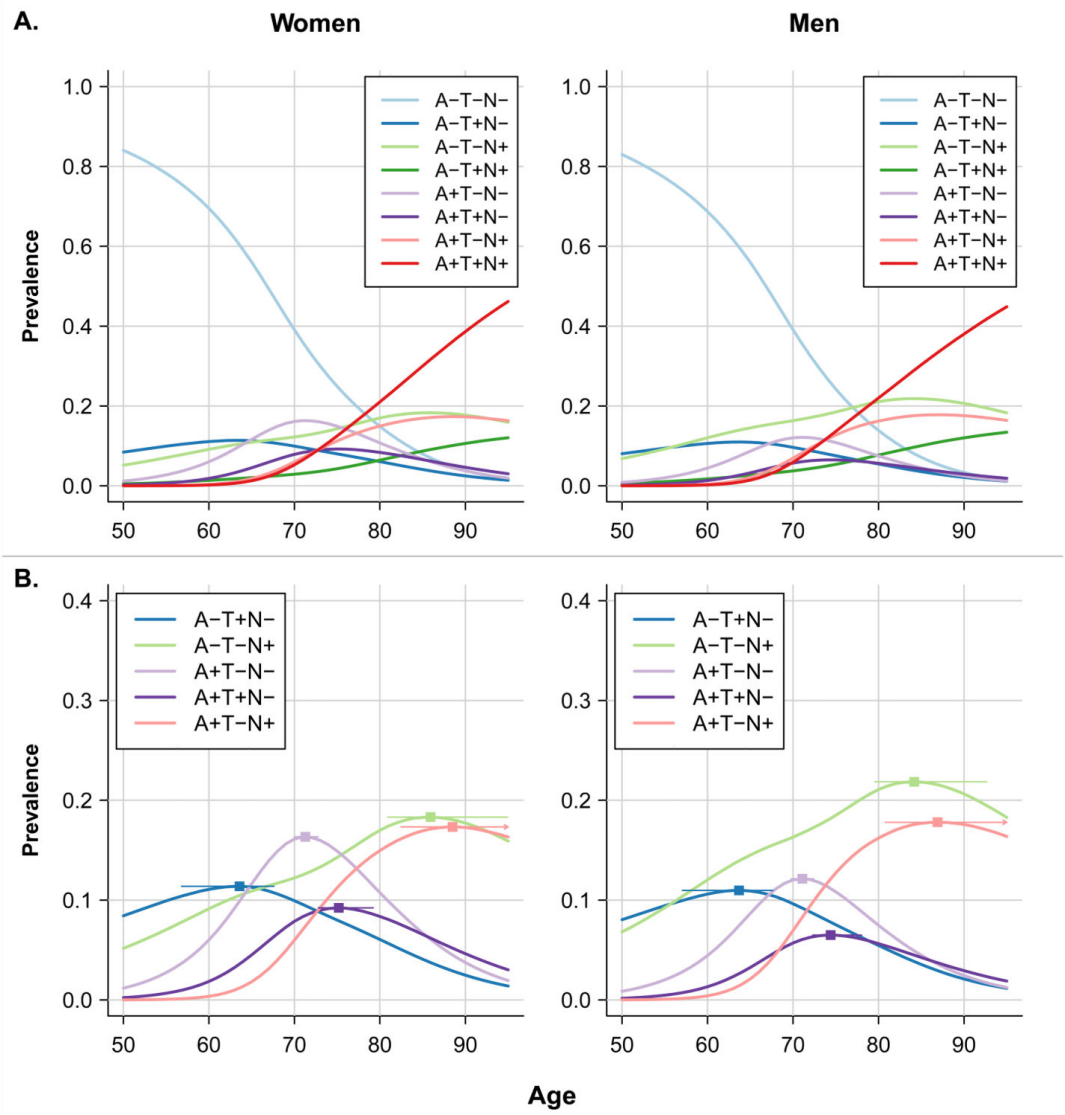
Estimated prevalence of the ATN biomarker groups by age and sex Panel A shows the estimated prevalence curves by age and sex for all ATN groups. Panel B shows the same curves as panel A (except for the A−T−N−, A−T+N+, and A+T+N+) on an enlarged scale with the estimated peak for each curve shown with a square and a 95% confidence interval.

For both men and women, A−T−N− prevalence declines from age 50 onward while A−T+N+ increases gradually with age starting at 60 and A+T+N+ increases more markedly with age beginning in the late 60s (Fig 2A) All of the remaining ATN groups reach individual peaks in prevalence.

Within an ATN group, comparisons of the curves for men versus women (Fig S2) reveal slightly greater prevalence of A−T−N+ in men from age 65-75 but no other clear sex differences.

We averaged the sex-specific prevalence estimates in order to make direct age-specific prevalence comparisons between ATN groups (Fig S3). The dominant trends are: A−T−N− is the most prevalent group from age 50 to the late 70s. From the early 80s onward, A+T+N+ is the most prevalent group.

All groups except A−T−N−, A−T+N+, and A+T+N+ reach a peak prevalence. The age at which the prevalence curve peaks differs considerably among ATN groups but is similar for men and women within each group (Table 2, Fig 2B). A−T+N− is the first group to peak (age 64) followed by A+T−N− and A+T+N− (ages 71 and about 75, respectively). The N−groups (A−T+N−, A+T−N−, and A+T+N−) all peak by age 75 or earlier while the N+ groups (A−T−N+ and A+T−N+) do so at or above age 84. Table S2 shows pairwise comparisons of peak ages between the ATN groups. Differences in peak age between some ATN groups are substantial, particularly between N−and N+ groups. For example the A+T−N+ and A−T−N+ groups peak 24.9 and 21.8 years later than the A−T+N− group.

**Figure 3:**
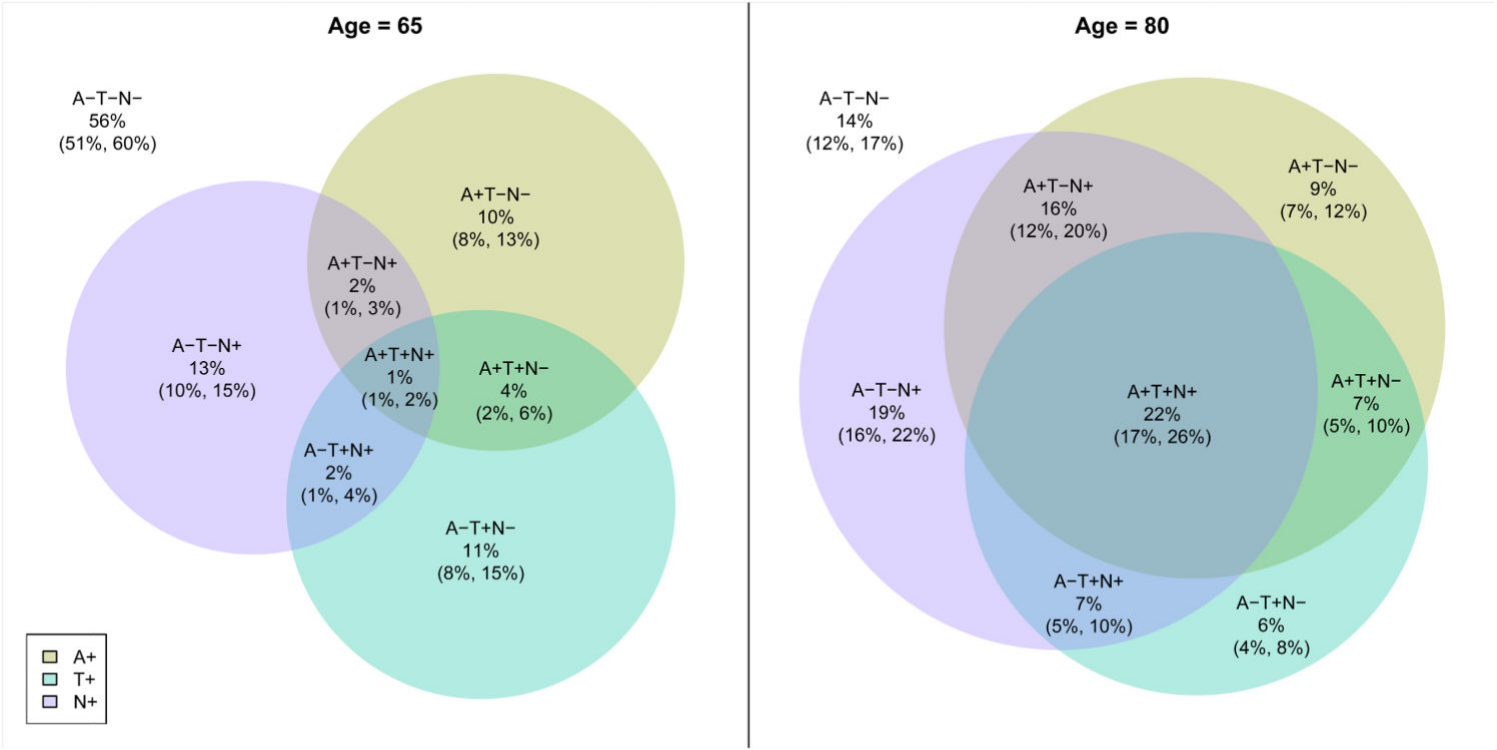
Venn diagram of the estimated prevalence of each ATN group at age 65 and age 80 These estimates are averaged over men and women. Since estimates are for a given age among clinically normal individuals, weighting to the population is not necessary. 95% confidence intervals for the estimates are also shown.

Fig 3 illustrates the proportions of individuals at ages 65 and 80 who have abnormal A, T, and N. This figure illustrates that abnormalities in these three biomarkers mostly do not overlap with each other at young ages. At older ages, the presence of more than one abnormal biomarker is common and there is considerable discordance among the three biomarkers.

ATN prevalence by age was also computed using HVa instead of cortical thickness as the N measure (Fig S4). While, agreement between the HVa and thickness measures was moderate (kappa = 0·45), overall the ATN prevalence trends by age were similar when either HVa or cortical thickness was used. One notable difference was a higher prevalence of N+ in men than women when using HVa, which is evident when comparing the A−T−N+ curves between men and women (Fig 2A vs S4).

## Discussion

Our main findings were the following. A−T−N− prevalence declines from age 50 onward while A−T+N+ and A+T+N+ increase continuously with age for both men and women. A−T−N− is the most prevalent group from age 50 to the late 70s. From the late 70s onward, A+T+N+ is the most prevalent group. The N− groups (A−T+N−, A+T−N−, and A+T+N−) all reached a peak prevalence by age 75 or earlier while the N+ groups (A−T−N+ and A+T−N+) reached a peak prevalence at or above age 84. .

Cross-sectional prevalence curves are a first step in understanding the complex and interdependent evolution of amyloid, tau, and neurodegeneration in aging individuals. Because our sample comes from a geographically stable population secular changes are likely to be minimized as much as possible, and thus we interpret differences in ATN prevalence curves across the 50 to 90 age range as being largely due to transitions between biomarker groups as people age. The declining prevalence of A−T−N− with age is logical since individuals can only transition out of A−T−N−, while the increasing prevalence of A+T+N+ with age makes sense because this is an absorbing biomarker state – i.e. people who remain CN can transition out of all states except A+T+N+ (Fig 2A). Interestingly, the increasing prevalence of A– T+N+ may reflect an absorbing state for those on a non- AD pathway.

For the other five ATN groups, the prevalence increases to a peak with age and then declines. The age at which the prevalence curves peak differs considerably among ATN groups (table 2, table S2, Fig 2B), but peak ages can be grouped into two clusters. The N– groups with evidence of either abnormal amyloid or tau deposition (A– T+N–, A+T– N–, and A+T+N–) all peak by age 75 or earlier while the N+ groups (A– T– N+ and A+T– N+) peak at or above age 84. From age 75 – 85 the prevalence of these three N– groups falls while the prevalence of these two N+ groups rises. This is consistent with the idea that neurodegeneration/neuronal injury is a downstream consequence of antecedent proteinopathies. The fact that A−T−N+ is more frequent than some N −groups in middle age (Fig S3) is consistent with the idea that this group is on a separate, non-AD trajectory where neurodegeneration is not driven by AD proteinopathy.

Overall the effect of sex on the prevalence of all ATN groups is minimal when using cortical thickness (Fig 2A, Fig S2), but more pronounced when using HVa (Fig S4).

APOE4 was more frequent among the A+ than the A− groups (Table 1, Fig 1). Among those who were A-, we found no clear evidence of elevation in APOE e4 frequency among A−T+N−, A−T−N+, or A−T+N+ relative to A−T−N− (Fig 1, table 1). Similarly, among those who are A+, we found no evidence of elevation in APOE ε4 frequency among A+T+N−, A+T−N+, or A+T+N+ relative to A+T−N−. One interpretation of this is that the primary effect of APOE ε4 is to increase amyloidosis, not to enhance tau deposition, neurodegeneration, or both through non-amyloid related mechanisms.

Abnormal biomarker profiles are associated with worse cognition across different domains after adjusting for age (Fig 1, Fig S1, table 1). WMH volume was higher in N+ in comparison to N− groups (p=0·05) (Fig 1, Fig S1, table 1). This supports the position that ischemic brain injury is, among other conditions, ^29^a likely contributor to N+.

SNAP was first described as A−N+ where N+ was based on FDG PET and MRI findings.^7^ In the 2011 NIA-AA criteria, the definition of N+ also included abnormal CSF phosphorylated and total tau.^10, 11^ In our present data, 15% of individuals were classified as SNAP defined by MR and amyloid PET at age 65, and 26% at age 80. Of these, 13% at age 65 and 27% at age 80 also had abnormal tau PET (i.e. A−T+N+) (Fig 3). Thus, tau and neurodegeneration are concordant only in a minority of A−N+ (SNAP) individuals where N+ is defined cortical thickness.^30, 31^ Mormino et al ^32^ and Wisse et al ^32^ reported that tau was not elevated in SNAP relative to A−N− individuals who were classified by amyloid PET and hippocampal volume and/or FDG PET using the NIA-AA plus SNAP 4-group construct. Similarly, we found that the proportions of T+ participants were similar among the A−N−and A−N+ groups, (16% vs 13% at age 65 and 30% vs. 27% at age 80) (Fig 3). However, by classifying A, T and N separately, we demonstrated that tau PET is frequently abnormal in SNAP where N+ is defined by cortical thickness. Tau PET had not yet been studied in humans when SNAP was first described. If the A−T+N−profile is included in the SNAP category where T+ is defined by tau PET, which we believe should be the case, then the proportion of SNAP with evidence of tauopathy is 50% at age 65 and 41% at age 80 (Fig 3).

We postulate that the A−T−N+ profile corresponds to neurodegeneration due to a heterogeneous group of non- AD pathologies that increase in prevalence with age including cerebrovascular disease, Lewy body disease,TDP 43, argyrophilic grains, and hippocampal sclerosis.^34^ A logical assumption is that the A−T+N−profile corresponds to primary age related tauopathy (PART).^35^ The A−T+N+ profile may correspond to a combination of PART and the other non-AD pathologies mentioned above. However, imaging - autopsy correlation studies will be needed confirm these hypotheses.

The four A+ profiles represent preclinical AD by the 2011 NIA-AA guidelines. A+T−N− corresponds to NIA- AA preclinical AD stage 1. A+T+N−, A+T−N+, and A+T+N+ all correspond to NIA-AA preclinical AD stage 2/3. Thus, tau and neurodegeneration are discordant in the majority of NIA-AA preclinical AD stage 2/3 individuals at age 65 (86%) and in half at age 80 (51%) (Fig 3).^30,31^ A model of AD pathogenesis proposes that amyloidosis promotes increased local tau deposition and its spread, which in turn is responsible for neurodegeneration. The ATN biomarker counterpart would be a sequence of A+T−N− to A+T+N− to A+T+N+. The facts that the median ages of these three groups (Table 1, Fig 1) and that the ages at which the prevalence curves peak (table 2, table S2, Fig 2B) increase incrementally lends support to the idea that A+T −N−to A+T+N− to A+T+N+ is the biomarker sequence of preclinical AD. However, longitudinal data will be necessary to confirm this chronological sequence. he A+T−N+ profile, which does not fit into the sequence of preclinical AD proposed above, perhaps indicates individuals in whom two different types of pathologies are evident by biomarkers: a non-AD degenerative process(es) resulting in N+, plus early AD resulting in the A+T−profile.

For our primary analyses, we used cortical thickness rather than commonly used hippocampal volume as our measure of neurodegeneration to avoid necessitating an adjustment for head size. Brain volumes scale with head size and correcting for this is not straightforward since head size is related to sex, yet sex-specific effects on atrophy likely exist. A solution is to use cortical thickness which does not scale closely with head size and consequently does not require an adjustment.^37^ Overall, the ATN prevalence curves by age were similar when either HVa (Fig S4) or cortical thicknesses (Fig 2A) were used as the N measure. These findings suggest that the ATN prevalences we report should be robust to different definitions of N. However, with only moderate agreement between abnormal HVa and thickness, there may be differences in which individuals are labeled N+ by the two biomarkers. We are uncertain if the more pronounced sex differences when using HVa as the N measure represent an artifact of head size adjustment or a true biological effect.

Our operationalization of the ATN scheme reflected a number of methodological factors and decisions. Both clinical- imaging correlation^14-18^ and autoradiographic ^38,39^ evidence, points to AV1451 as a useful measure of the 3R/4R paired helical filament tau deposits that are characteristic of AD and primary age related tauopathy ^35^.Binding in primary tauopathies (except those that produce 3R/4R fibrillar tau deposits) is less certain. In this study, we used a single reporter tau PET meta ROI that included medial, basal, and lateral temporal lobe areas 23.Our rationale was that tau PET uptake in these areas is consistently associated with characteristics of AD such as the presence of amyloid on PET, worse cognitive performance across the clinical spectrum, and abnormal CSF phosphorylated tau 14-18. This set of ROIs captures a broad dynamic range across the normal to pathologic aging to AD dementia spectrum; it therefore seems to represent a reasonable tau PET summary reporter ^23^.^23^

The ATN framework requires defining abnormality in each biomarker. We previously conducted a thorough examination of different methods for selecting cut-points to define abnormality on amyloid PET, tau PET and cortical thickness.^23^ We regard plaques, tangles and synapse loss to be pathological. While all of these processes increase in frequency and severity with age ^34^, our cut-points were not age-adjusted. Our position is that while not age-norming the cut-points results in a greater proportion of older individuals being labeled abnormal, the fact that an entity is frequent does not disqualify it from being pathological. While age-norming cognitive tests is a common practice, biomarkers in other fields are typically not age-normed. For example the cut-points used to define diabetes or hypertension are not changed with age. Loss of synapses and dendritic spines and associated cognitive/functional loss seems to be a nearly universal feature of aging in humans and a range of animal species. ^40, 41^ Whether this should be considered pathological or not is an unresolved question.

The methods of selecting reporter meta ROIs and cut-points used in ATN classification were AD-centric. However, while temporal lobe atrophy is characteristic of AD, it is not diagnostic for AD. A variety of non-AD conditions (argyrophillic grains, hippocampal sclerosis, etc.) may produce atrophy in these brain areas. However, until specific biomarkers of the common non-AD entities are developed, the only available biomarker evidence of their presence is nonspecific indicators of neurodegeneration/neuronal injury.

Our study has limitations. Because eight possible ATN combinations exist, participant numbers in some groups are small. Dichotomizing each biomarker simplifies what is an underlying continuous process. Measurement imprecision will inevitably result in some classification errors particularly for values close to cut-points. With three different biomarker classes per individual, the likelihood of classification error is compounded compared to a situation where only a single biomarker is used. We have not examined individuals in the population who have become clinically impaired; this awaits greater enrollment of impaired individuals in the MCSA. While the most rational explanation for the observed changing ATN prevalances with age is within-subject ATN group transitions, our data are cross-sectional. Our study raises interesting questions for which no answers exist at this time. For example, what are the longitudinal clinical/cognitive outcomes and the pathological underpinnings of these ATN groups? Answers to these questions require longitudinal clinical follow-up in large numbers of well characterized individuals with eventual autopsy correlation. To our knowledge, these data do not exist anywhere at this time for individuals characterized by ATN profile. Data addressing these issues will await maturation of our and other research cohorts.

## Supplementary Material

supplement **Figure S1. Age adjusted plots of WMH volume and cognition by ATN grou**Box plots of partial residuals of the log of WMH volume and cognitive z-scores after regressing out the effect of age. These plots are weighted to the clinically normal Olmsted County population by age and sex.
**Figure S2. Estimated prevalence with 95% confidence limits of the ATN biomarker groups by age and sex.** Since estimates are for a given age and sex among clinically normal individuals, weighting to the population is not necessary. Differences between men and women within biomarker group are also shown where values above zero indicate ages where the biomarker prevalence is higher in men than women and values below zero where the biomarker prevalence is higher in women than men. Differences are considered statistically significant if the confidence limits do not include zero.
**Figure S3. Pairwise differences in prevalences among ATN biomarker groups**. Values above zero indicate ages where the biomarker prevalence for the first group listed in the title is higher than in the second group and values below zero where the biomarker prevalence is higher for the second group listed in the title than the first. Differences are considered statistically significant if the confidence limits do not include zero. These curves were averaged over men and women.
**Figure 4S. Estimated prevalence of the ATN biomarker groups by age and sex where N is defined using hippocampal volume adjusted for head size**. Since estimates are for a given age and sex among clinically normal individuals, weighting to the population is not necessary.

## Figures and Tables

**Table 1:** Characteristics of participants by ATN biomarker classification

Characteristic	A−T−N− n = 165	A−T+N− n = 35	A−T−N+ n = 63	A−T+N+ n = 19	A+T−N− n = 44	A+T+N− n = 25	A+T−N+ n = 35	A+T+N+ n = 49
Age, years	38%	8%	14%	4%	10%	6%	8%	11%
Median (IQR)	65 (58, 69)	68 (62, 78)	75 (67, 81)	79 (73, 82)	71 (66, 78)	77 (70, 82)	82 (77, 85)	82 (76, 87)
Min, Max	51, 84	53, 90	53, 90	63, 95	53, 88	65, 94	66, 91	64, 94
Male gender, no. (%)	83 (50%)	19 (54%)	45 (71%)	9 (47%)	16 (36%)	15 (60%)	22 (63%)	27 (55%)
Education, years, Median (IQR)	16 (13, 16)	16 (14, 17)	15 (13, 16)	16 (13, 16)	14 (13, 17)	14 (14, 17)	14 (12, 16)	14 (12, 16)
APOE ε4 positive, no. (%)	30 (19%)	5 (15%)	13 (22%)	1 (5%)	20 (49%)	13 (52%)	14 (41%)	16 (33%)
WMH volume, Median (IQR)	5.3 (3.4, 9.7)	6.8 (3.7, 10.4)	11.9 (5.6, 17.3)	15.0 (8.2, 20.7)	9.6 (5.2, 15.5)	8.9 (6.4, 16.0)	19.1 (9.7, 33.1)	18.9 (11.1, 31.7)
Cognitive z-scores, Median (IQR)								
Memory	0.5 (-0.2, 1.0)	-0.1 (-0.5, 0.7)	-0.2 (-0.8, 0.6)	0.2 (-0.8, 0.9)	0.1 (-0.5, 0.5)	0.1 (-0.8, 0.8)	-0.6 (-1.1, -0.1)	-0.5 (-1.1, 0.3)
Attention	0.4 (-0.1, 0.9)	0.6 (0.0, 0.9)	-0.2 (-0.8, 0.3)	0.2 (-0.2, 0.8)	0.1 (-0.5, 0.6)	-0.2 (-0.7, 0.3)	-0.3 (-0.8, 0.2)	-0.5 (-1.4, 0.0)
Language	0.3 (-0.3, 0.9	0.5 (-0.2, 1.1)	-0.2 (-0.7, 0.3)	-0.0 (-0.4, 0.4)	0.2 (-0.2, 0.8)	0.0 (-0.6, 0.4)	-0.6 (-1.0, 0.0)	-0.3 (-0.8, 0.1)
Visuospatial	) 0.3 (-0.3, 0.9)	0.3 (-0.2, 0.8)	-0.2 (-0.7, 0.4)	0.1 (-0.4, 0.5)	0.1 (-0.8, 0.5)	0.2 (-0.6, 0.7)	-0.4 (-0.8, -0.0)	-0.1 (-1.3, 0.4)
Amyloid PET, Median (IQR)SUVR	1.31 (1.26, 1.35)	1.33 (1.30, 1.37)	1.33 (1.28, 1.37)	1.35 (1.31, 1.37)	1.57 (1.47, 1.77)	1.62 (1.55, 2.10)	1.58 (1.50, 1.77)	2.22 (1.54, 2.44)
Centiloid	9 (5, 12)	11 (8, 14)	11 (7, 14)	13 (9, 14)	31 (23, 48)	35 (29, 76)	32 (25, 48)	86 (28, 105)
Tau PET, SUVR, Median (IQR)	1.15 (1.11, 1.19)	1.28 (1.25, 1.30)	1.17 (1.10, 1.20)	1.28 (1.25, 1.30)	1.16 (1.13, 1.20)	1.27 (1.25, 1.34)	1.16 (1.12, 1.20)	1.30 (1.26, 1.36)
Cortical thickness, mm, Median(IQR)	2.79 (2.73, 2.85)	2.78 (2.75, 2.87)	2.59 (2.53, 2.62)	2.59 (2.52, 2.62)	2.76 (2.72, 2.82)	2.76 (2.72, 2.78)	2.59 (2.47, 2.63)	2.56 (2.51, 2.62)

**Table 2:** Age (95% CI) at which the percentage of each ATN prevalence curve reaches its peak among women and men Differences in peaks by sex are also shown.

Group	Women Peak Age (95% CI)	Men Peak Age (95% CI)	Men vs. Women Diff. Peak Age (95% CI)
A−T+N−	64 (57, 68)	64 (57, 68)	0.1 (-1.2, 1.8)
A+T−N−	71 (70, 73)	71 (70, 72)	-0.2 (-1.0, 0.4)
A+T+N−	75 (73, 79)	74 (72, 78)	-0.8 (-2.2, 0.3)
A−T−N+	86 (81, 95)	84 (80, 93)	-1.8 (-3.9, 0.4)
A+T−N+	88 (82, 100)	87 (81, 100)	-1.6 (-3.8, 0.9)

## Research in Context

### Evidence before this study

We searched PubMed with the terms “preclinical AD”, “tau PET”, and “amyloid PET” from January 2006 - September 2016, English language only. Clinically normal cohorts have been studied using the NIA-AA staging plus SNAP construct resulting in four different biomarker categories: A−N−, A+N−, A−N+ (SNAP) or A+N+. Proportions of these four groups were roughly similar in many cohorts. APOE ε4 was much more common in A+N− and A+N+ than in A−N− or SNAClinical and psychometric outcomes were uniformly worst in A+N+. These findings were largely the same whether biomarker categorization was done using imaging or CSF. The NIA-AA staging plus SNAP construct has been useful because it provided a common framework for different research groups to communicate their own findings.

### Added value of this study

In retrospect, a weakness of the NIA-AA staging plus SNAP construct is the grouping of CSF phosphorylated tau into the same neurodegeneration/neuronal injury category with total tau, MRI and FDG PET. The ATN construct remedies this weakness and enables researchers to investigate multi-domain biomarker associations where the effects of tauopathy (defined by tau PET or CSF phosphorylated tau) and neurodegeneration/neuronal damage (defined by CSF total tau, MRI and FDG PET) at the individual level are segregated. We describe clinical characteristics and age- and sex- specific prevalences of individuals age 50 and older using the ATN construct. To our knowledge, this is the first study to do so. We found that tau and neurodegeneration were often discordant. Among the individuals in our sample who would be labeled NIA-AA stage 2/3 preclinical AD (i.e. A+T+N−, A+T−N+, and A+T+N+), 86% are discordant between T and N at age 65 and 51% at age 80. Among the individuals in our sample who would be labeled SNAP (i.e. A−T+N,− A−T−N+, A−T+N+), 92% are discordant between T and N at age 65 and 78% at age 80.

### Implications of all the available evidence

The ATN classification scheme is a useful approach to biomarker characterization with the goal of more fully understanding the underlying heterogeneous pathology in the population. Marked age variation in prevalences requires careful interpretation of biomarker results from studies across cohorts of different ages. Future research will elucidate the within-subject biomarker changes to evaluate amyloid dependent (i.e. AD) and amyloid independent (i.e. SNAP) pathological pathways and sequences of biomarker abnormality.
