# Supplementary material for: Age and sex specific prevalences of cerebral β-amyloidosis, tauopathy and neurodegeneration among clinically normal individuals aged 50-95 years: a cross-sectional study
Source: Lancet Neurol. Author manuscript; Available in PMC 2018 Jun 1. (PMC5516534; doi:10.1016/S1474-4422(17)30077-7)
Supplement: supplement — Figure S1. Age adjusted plots of WMH volume and cognition by ATN grouBox plots of partial residuals of the log of WMH volume and cognitive z-scores after regressing out the effect of age. These plots are weighted to the clinically normal Olmsted County population by age and sex. Figure S2. Estimated prevalence with 95% confidence limits of the ATN biomarker groups by age and sex. Since estimates are for a given age and sex among clinically normal individuals, weighting to the population is not necessary. Differences between men and women within biomarker group are also shown where values above zero indicate ages where the biomarker prevalence is higher in men than women and values below zero where the biomarker prevalence is higher in women than men. Differences are considered statistically significant if the confidence limits do not include zero. Figure S3. Pairwise differences in prevalences among ATN biomarker groups. Values above zero indicate ages where the biomarker prevalence for the first group listed in the title is higher than in the second group and values below zero where the biomarker prevalence is higher for the second group listed in the title than the first. Differences are considered statistically significant if the confidence limits do not include zero. These curves were averaged over men and women. Figure 4S. Estimated prevalence of the ATN biomarker groups by age and sex where N is defined using hippocampal volume adjusted for head size. Since estimates are for a given age and sex among clinically normal individuals, weighting to the population is not necessary. [file NIHMS873273-supplement.pdf]

Supplementary Methods, Tables, and Figures

1. Supplementary Statistical Methods

Because the Mayo Clinic Study of Aging (MCSA) study design oversampled individuals in the oldest ages, overall summaries by ATN group which aggregate over age and sex have been weighted to match the age and sex distribution of clinically normal individuals in Olmsted County, Minnesota. The weights were obtained as follows. Census Bureau estimates for 2010 provided the age and sex distribution of the population and published prevalence rates of MCI and dementia in Olmsted County were used to get the fraction of the population that was clinically normal in each 5-year age and sex strata.[1,2]

Results that have been weighted to the population are found in Figure 1 and Supplementary Figure 1. Statistical significance for these results were based on survey sampling methods used in the *svyglm()* function in the contributed *survey* package in the R statistical software suite. The results that display unweighted data or which explicitly condition on age and sex do not need to be reweighted. This applies to all other figures and tables.

Imaging visits from 1548 MCSA individuals were used to estimate the prevalences of the eight classes by age and sex; tau data is available for 435 of these 1548 individuals. Let Pr(ATN | age, sex) be the age and sex specific prevalence, where ATN is one of the 8 ATN groups. This can be factored into two terms as Pr(AN | age, sex) Pr(T | AN, age, sex) where AN is one of the four AN groups and T is abnormal/normal tau. Because the missingness of tau is a function only of calendar time (since tau-PET scanning was added to the study later than other imaging modalities), the values are missing completely at random (MCAR).[3] This allows us to use all 1548 individuals to estimate the first term, and the 435 individuals with tau data to estimate the second term. Both terms were estimated (separately) using a multinomial regression model. For the second term the multinomial model is equivalent to logistic regression since the outcome (abnormal versus normal tau) is binary. Age and sex are covariates in the first model; age, sex and AN status are covariates in the second. Age is modeled with a restricted cubic spline with knots at ages 60, 70, and 80 in the first model and as a linear term in the second model. The approach is closely related, for this data set, to the EM algorithm solution to this problem proposed by Dempster et al.[4] Confidence intervals for the curves are based on resampling from the multivariate distribution of the parameters.[5]

References

1 Petersen RC, Roberts RO, Knopman DS, Geda YE, et al. Prevalence of mild cognitive impairment is higher in men. The Mayo Clinic Study of Aging. *Neurology* 2010; **75**(10):889–897.

2 Knopman DS, Petersen RC, Rocca WA, Larson EB, Ganguli M. Passive case-finding for Alzheimer’s disease and dementia in two U.S. communities. *Alzheimers Dement* 2011; **7**(1):53–60.

3 Dempster AP, Laird NM, Rubin DB. Maximum Likelihood from Incomplete Data via the EM Algorithm. Journal of the Royal Statistical Society Series B (Methodological) 1977; **39**(1):1–38.

4 Little RJA, Rubin DB. *Statistical Analysis with Missing Data*. 2nd ed. Hoboken, N.J: Wiley; 2002.

5 Gelman A, Hill J. *Data analysis using regression and multilevel/hierarchical models*. New York: Cambridge University Press; 2007.

2. Supplementary Tables

Characteristic	A−N−<br>n = 689<br>45%	A−N+<br>n = 313<br>20%	A+N−<br>n = 210<br>14%	A+N+<br>n = 336<br>22%
**Age, years**				
**Median (IQR)**	65 (58, 72)	77 (69, 83)	73 (68, 79)	82 (77, 86)
**Min, Max**	51, 92	53, 97	53, 94	61, 96
**Male gender, no. (%)**	347 (50%)	186 (59%)	93 (44%)	191 (57%)
**Education, years, Median (IQR)**	16 (13, 17)	14 (12, 16)	14 (12, 16)	14 (12, 16)
**APOE ε4 positive, no. (%)**	148 (22%)	45 (15%)	98 (48%)	117 (35%)
**Tau data, no. (%)**	200 (29%)	82 (26%)	69 (33%)	84 (25%)

Supplementary Table 1. Characteristics of MCSA individuals by AN biomarker classification

	A+T−N−<br>Diff (95% CI)	A+T+N−<br>Diff (95% CI)	A−T−N+<br>Diff (95% CI)	A+T−N+<br>Diff (95% CI)
**A−T+N−**	8.1 (3.1, 14.8)	11.8 (8.4, 16.8)	21.8 (14.2, 34.3)	24.9 (15.1, 42.3)
**A+T−N−**		3.7 (1.4, 7.4)	13.7 (9.1, 22.6)	16.8 (11.0, 27.4)
**A+T+N−**			10.0 (3.2, 20.0)	13.1 (4.7, 25.9)
**A−T−N+**				3.1 (-7.8, 13.3)

Supplementary Table 2. Pairwise differences in peak ages (with 95% CI) among the biomarker groups averaged among men and women.

3. Supplementary Figures

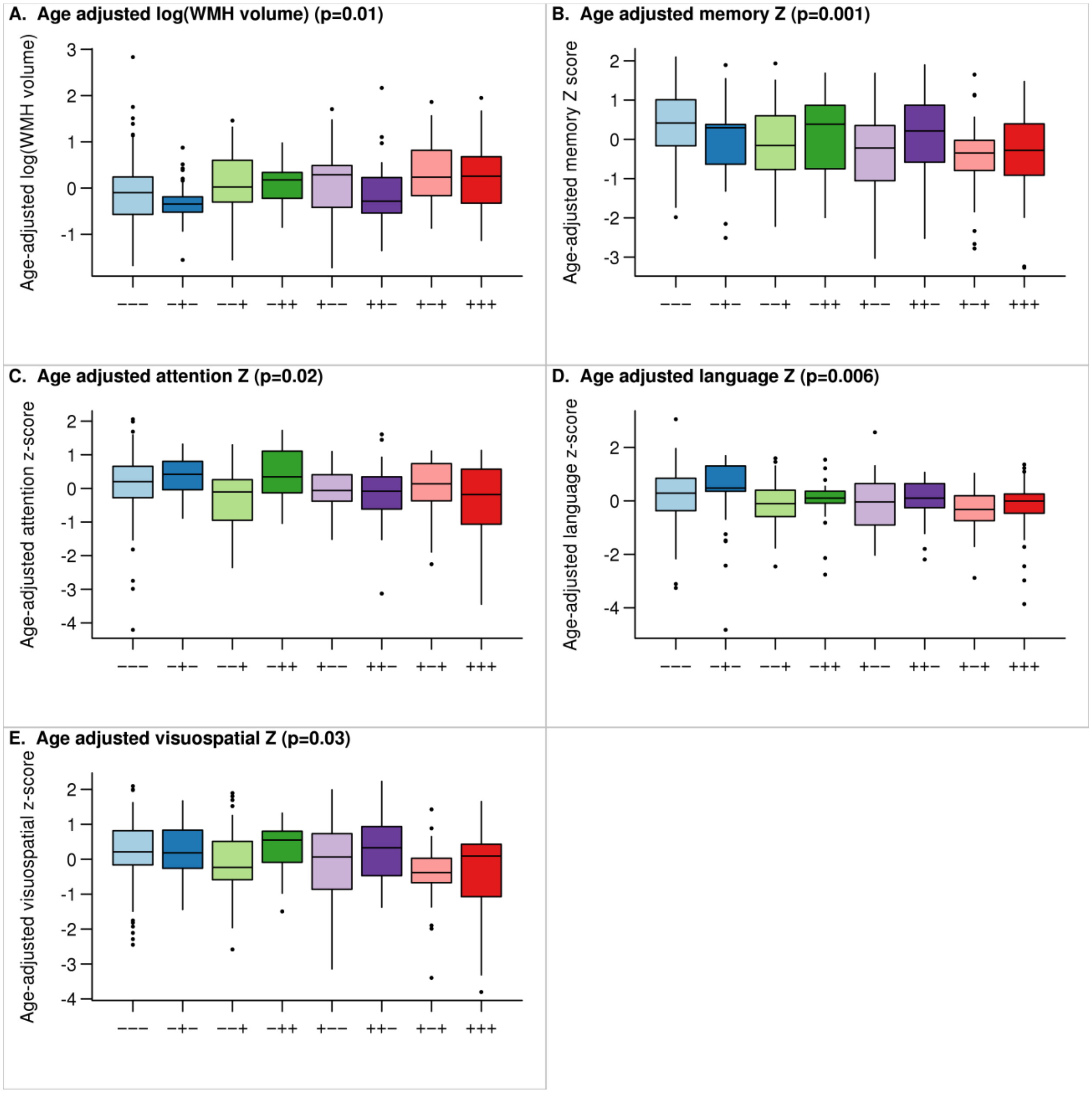

Supplementary Figure 1. Age adjusted plots of WMH volume and cognition by ATN group. Box plots of partial residuals of the log of WMH volume and cognitive z-scores after regressing out the effect of age. These plots are weighted to the clinically normal Olmsted County population by age and sex.

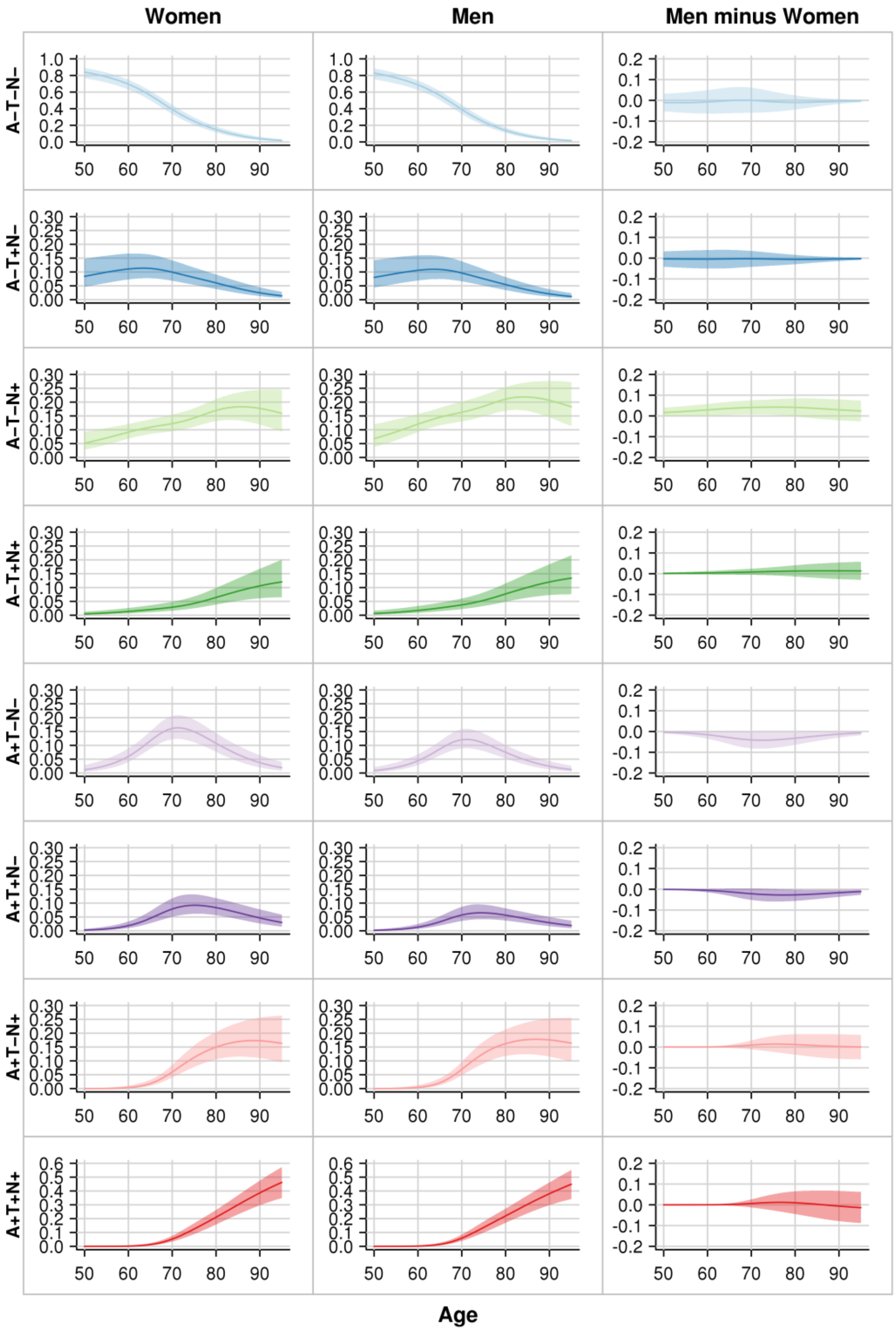

Supplementary Figure 2. Estimated prevalence with 95% confidence limits of the ATN biomarker groups by age and sex. Since estimates are for a given age and sex among clinically normal individuals, weighting to the population is not necessary. Differences between men and women within biomarker group are also shown where values above zero indicate ages where the biomarker prevalence is higher in men than women and values below zero where the biomarker prevalence is higher in women than men. Differences are considered statistically significant if the confidence limits do not include zero.

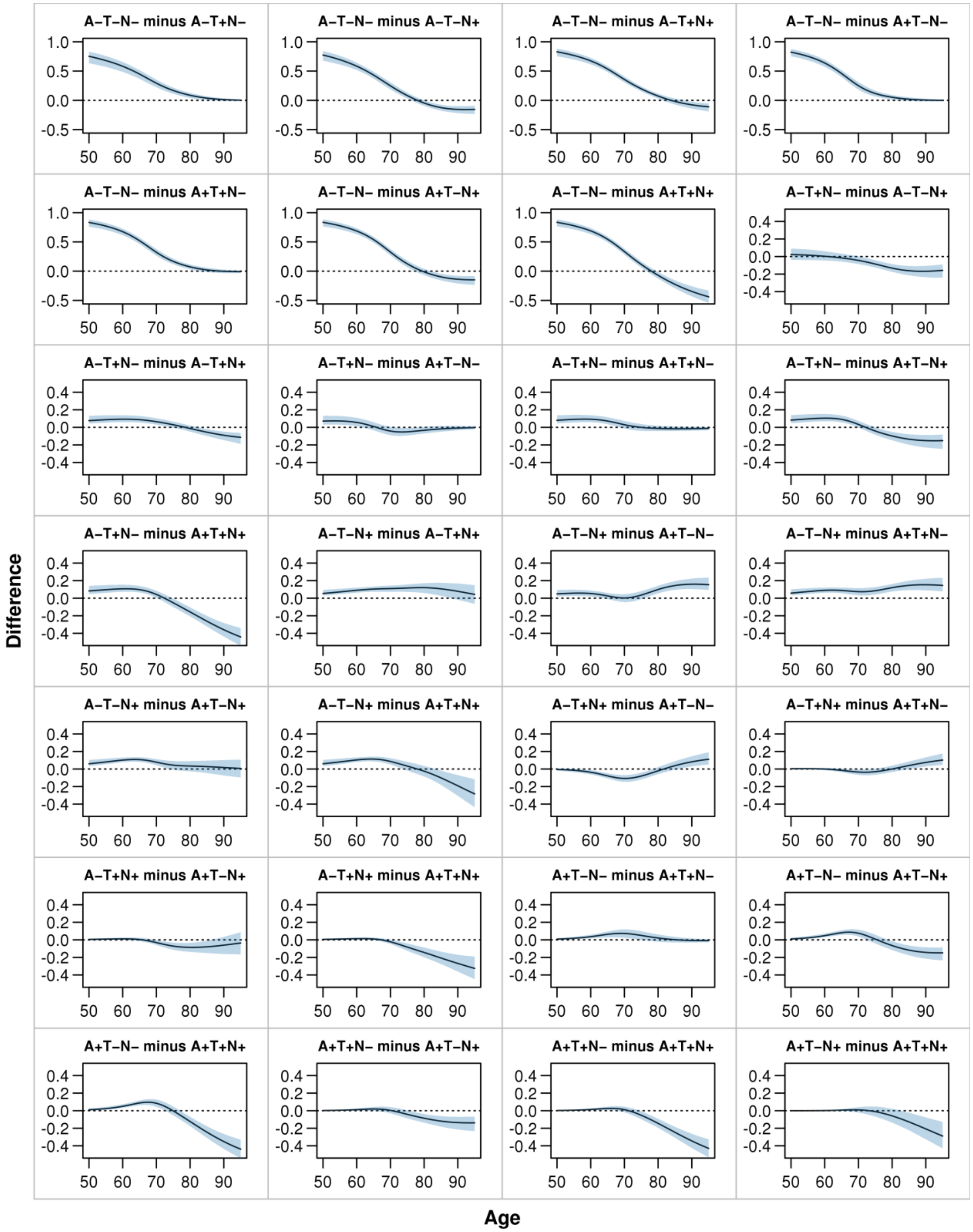

Supplementary Figure 3. Pairwise differences in prevalences among ATN biomarker groups. Values above zero indicate ages where the biomarker prevalence for the first group listed in the title is higher than in the second group and values below zero where the biomarker prevalence is higher for the second group listed in the title than the first. Differences are considered statistically significant if the confidence limits do not include zero. These curves were averaged over men and women.

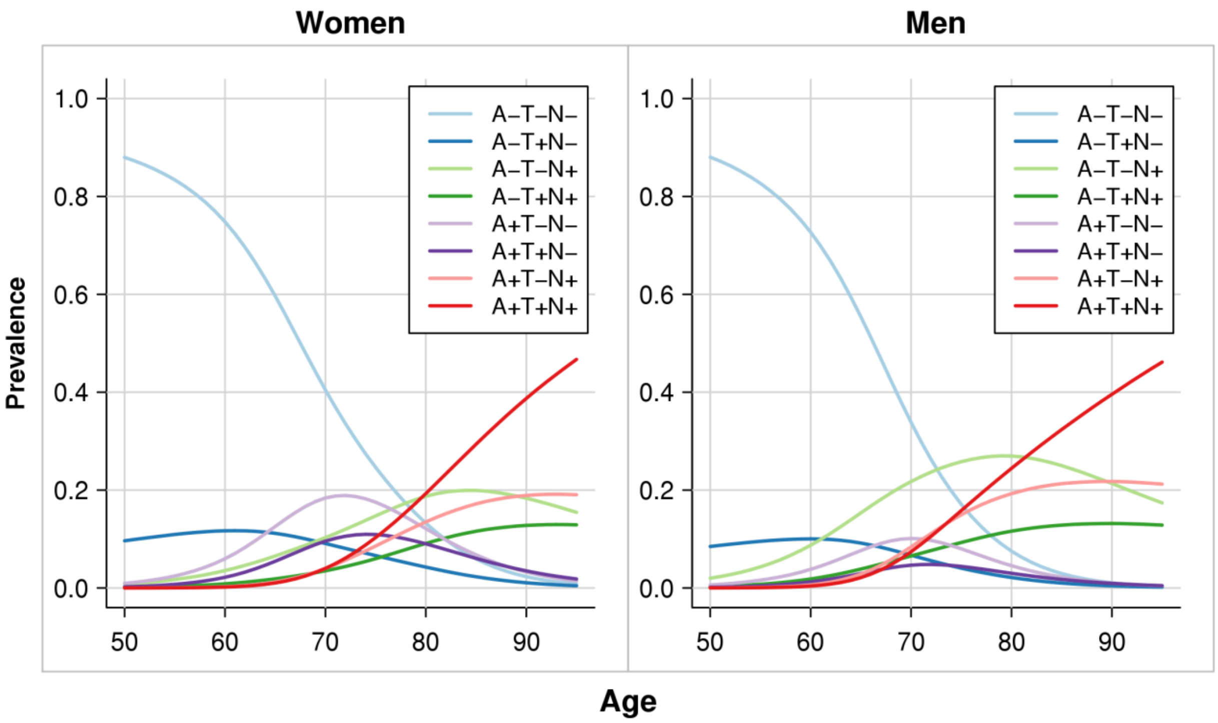

Supplementary Figure 4. Estimated prevalence of the ATN biomarker groups by age and sex where N is defined using hippocampal volume adjusted for head size. Since estimates are for a given age and sex among clinically normal individuals, weighting to the population is not necessary.
